# Working relationships between obstetric care staff and their managers: a critical incident analysis

**DOI:** 10.1186/s12913-016-1694-x

**Published:** 2016-08-26

**Authors:** Effie Chipeta, Susan Bradley, Wanangwa Chimwaza-Manda, Eilish McAuliffe

**Affiliations:** 1 College of Medicine-Centre for Reproductive Health, Private bag 360, Blantyre 3, Malawi; 2 Centre for Maternal and Child Health Research, School of Health Sciences, City University London, 1 Myddelton Street, London, EC1R 1UW UK; 3 School of Nursing, Midwifery and Health Systems, University College Dublin, Belfield, Ireland

**Keywords:** Working relationships, Leadership, Job satisfaction, Staff motivation, Work performance

## Abstract

**Background:**

Malawi continues to experience critical shortages of key health technical cadres that can adequately respond to Malawi’s disease burden. Difficult working conditions contribute to low morale and frustration among health care workers. We aimed to understand how obstetric care staff perceive their working relationships with managers.

**Methods:**

A qualitative exploratory study was conducted in health facilities in Malawi between October and December 2008. Critical Incident Analysis interviews were done in government district hospitals, faith-based health facilities, and a sample of health centres’ providing emergency obstetric care. A total of 84 service providers were interviewed. Data were analyzed using NVivo 8 software.

**Results:**

Poor leadership styles affected working relationships between obstetric care staff and their managers. Main concerns were managers’ lack of support for staff welfare and staff performance, lack of mentorship for new staff and junior colleagues, as well as inadequate supportive supervision. All this led to frustrations, diminished motivation, lack of interest in their job and withdrawal from work, including staff seriously considering leaving their post.

**Conclusions:**

Positive working relationships between obstetric care staff and their managers are essential for promoting staff motivation and positive work performance. However, this study revealed that staff were demotivated and undermined by transactional leadership styles and behavior, evidenced by management by exception and lack of feedback or recognition. A shift to transformational leadership in nurse-manager relationships is essential to establish good working relationships with staff. Improved providers’ job satisfaction and staff retentionare crucial to the provision of high quality care and will also ensure efficiency in health care delivery in Malawi.

**Electronic supplementary material:**

The online version of this article (doi:10.1186/s12913-016-1694-x) contains supplementary material, which is available to authorized users.

## Background

Malawi is currently facing a severe shortage of human resources for health as it operates with only 33 % of the health care workers needed to effectively deliver health care services to the population [1]. The nurse to population ratio is currently at 1:3,680 compared to the World Health Organisation’s recommended ratio of 1:1,000 [2]. Nurse/midwives provide the bulk of primary health care services, but many rural health centres’ have no full-time nurse or midwife despite the fact that 85 % of Malawi’s population resides in rural areas [3]. Delivery of maternal health care is a particular concern, with maternal mortality rates at 578 per 100,000 live births [4]. Working conditions for nurse/midwives have been characterized by infrequent supervision and support, high and uneven workloads, and inequitable access to training, which have all contributed to low morale and frustration [5–8]. The resulting loss of health care workers undermines health system capacity, as more experienced workers migrate and remaining staff face increased workloads, stress and demotivation, with obvious consequences for standards of care [9, 10].

A growing body of evidence demonstrates the importance of effective working relationships on nurses’ job satisfaction [11, 12]. The effect of job satisfaction on staff retention, patient satisfaction and organizational commitment has been documented [11, 13–15], while positive working relationships between nurses, their colleagues and managers, are among factors perceived to directly affect motivation and performance [16]. Studies have also shown the importance of interpersonal skills and managers’ relational competencies to improve the satisfaction of nurses. Communication, teamwork, conflict resolution and interpersonal skills are important management skills that can help improve manager/employee relationships, employee job satisfaction, quality care and work environments [17]. Chen et al. [18] also observed that work relationships between unit managers and staff promote organizational citizenship behaviours and can lead to low turnover intent in nurses. Further evidence relates the development of burnout to the interpersonal environment of the organization [19]; while organizational commitment to promote staff welfare is linked to decreased staff burnout.

Leadership practices are key determinants of staff motivation and organizational performance, with strong and supportive leadership as a strong predictor of staff motivation and morale [16, 20]. It has increasingly been recognized that transformational leadership is a predictor of quality outcomes in health care settings. The key dimensions of transformational leadership are idealized influence, inspirational motivation, intellectual stimulation and individual consideration [21]. This style of leadership involves stimulating followers to perform beyond the level of expectations [21], establishing leadership authority and integrity, and motivating and inspiring subordinates to pursue a shared vision of the future. These directly influence organizational citizenship behaviour and performance, staff well-being, patient safety and staff satisfaction [22]. Managers who use transformational leadership styles recognise and appreciate staff efforts, identify and reward good performance, ensure equal access to opportunities and promote good interpersonal relationships. These actions demonstrate that hospital management are interested in providing a good working environment and promoting staff welfare [23, 24]. This in turn influences staff motivation, increases job satisfaction and results in low staff turnover [25–28].

In contrast, the key elements of transactional leadership are contingent reward, management by exception and laissez-faire leadership [29]. Transactional leadership assumes that punishments and rewards are necessary to motivate people, leading to a manager-subordinate working relationship based on ‘give and take’ if staff meet expected levels of performance [21]. The leader’s focus is on monitoring how well tasks have been accomplished, identifying and correcting problems to maintain desired performance levels [29, 30]; often the leader avoids making any decisions at all. This leadership style is associated with increasedstaff dissatisfaction and turnover. Staff are left feeling unsupported due to a lack of meaningful interaction with their managers, whichinfluences staff decisions on whether or not to stay in their job [13, 25]. Staff burnout, job dissatisfaction, staff turnover and absenteeism are important variables in understanding the impact of transactional leadership on organizational performance. Analysis of these factors is crucial to understanding manager-staff relations and their impact on staff development and delivery of high quality care.

Poor or inefficient working relationships are associated with mistrust, chronic stress, and dissatisfaction among nurses, which have a negative impact on organisational performance. Attention to positive working relationships is essential for the development of work environments with low staff burnout or reduced turnover and also where safe and excellent care can be provided to patients and their families [31]. Recent studies on human resources for health in low-income settings have focused on motivation and retention of health care workers in general [13, 32–34]. The impact of health care manager and staff relationships on retention and performance has been mentioned in very few studies [24]. In Malawi, there is little evidence to highlight the impact of the working relationship between managers and nursing staff, despite reports that poor relationships cause distress [35] and many qualified nurses continue to leave the health service. Along with clinical officers, the enrolled and technician cadres of nurse/midwife are the mainstay of the Malawian health service at the primary care level [8, 36].

This study was part of a larger project, Health Systems Strengthening for Equity (HSSE), whose goal was to expand the evidence base in support of effective use of mid-level providers (MLPs) in maternal and neonatal health. HSSE aimed to increase recognition and effective use of MLPs, and advocate for an enabling environment that optimizes their performance in order to strengthen health systems. Improving human resource management (HRM) is a crucial issue for Malawi’s Ministry of Health (MOH). To date, there are limited data on health workers’ perceptions of the impact of their relationships with health facility managers on retention and performance. Understanding the work environment for nurses/midwives and how they relate with their managers is crucial to ensure that they are well supported and enabled to deliver a good standard of service, especially to the rural population. This can assist in the development of realistic strategies to retain nurse/midwives working in primary care facilities or districts and improve their performance. This study therefore sought to understand how nurse/midwives perceive their relationship with their managers.

## Methods

### Study design

This qualitative exploratory research study was part of the larger HSSE study. In-depth interviews with facility-based health care providers were done using Critical Incident Analysis (CIA). The critical incident technique [37] is a flexible tool that uses probing questions to elicit events that have particular importance for participants. This methodology is often used to explore events that respondents feel have been critical to their experience of their job [38].

### Study setting and population

This was a near national study that cut across Malawi and involved 25 of Malawi’s 28 districts. Participants were selected from government district hospitals, Christian Health Association of Malawi (CHAM) health facilities, and a sample of health centres providing emergency obstetric care. Information circulars were sent out to facilities a week in advance, inviting participation. Nurse/midwives were often located in the maternity unit, but maternity and/or facility in-chargesalso assisted in identifying other staff involved in obstetric care who were located elsewhere in the facility. A purposive sample was used to recruit eligible respondents. These had to have performed at least one of the Emergency Obstetric Care (EmOC) Signal Functions^1^ in the last 3 months and to have experienced an incident within those 3 months that had made them seriously consider leaving their job. A total of 84 eligible health care personnel within selected facilities participated in this study.

### Data collection

Data collection took place from October-December 2008. The CIA interview (Additional file 1) was an anonymous, semi-structured interview designed to identify specific incidents or moments of high salience that had a pivotal impact on a person’s experience of their job. To avoid confusing the word ‘critical’ with clinical emergencies or medical errors, participants were simply asked, *“In the past 3 months has there ever been a time when something happened to make you seriously consider leaving your job?”* A series of probing questions were then used to invite participants to:

- Describe the incident;
- Identify the events/factors leading up to it;
- Recount how the incident had made them feel;
- Describe any impact it had on their performance; and
- Discuss where they would go if they did leave the facility.

Most interviews were in English and were recorded and transcribed verbatim in Microsoft Word. A few were conducted in Chichewa, a common local language, at the participant’s request. These interviews were transcribed in Chichewa then translated into English by researchers who were fluent in both languages.

### Data analysis

Data were analyzed using NVivo 8 qualitative software [39] by a team of experienced researchers familiar with the context and the existing literature. A process of inductive and deductive coding was used to identify emerging themes [40]. The main emerging thematic areas were further expanded into sub-nodes during data analysis. Detailed descriptors of each node allowed the analysis teams to validate the content of nodes and discuss their coding and interpretations of the data in detail to improve inter-coder reliability and enhance dependability of the analysis.

## Results

### Demographics of the study population

Eighty-four health workers participated in this study. Table 1 outlines participating cadres as categorized in the Malawi Health Service Strategic Plan [36].

**Table 1:** Health worker participation in the study, by cadre

Cadre	Total	Female	Male
Nurse-Midwife Technicians (NMT)	40	35 (88 %)	5 (12 %)
Enrolled Nurse/Midwives (ENM)	12	12 (100 %)	0
Registered Nurse/Midwives (RN/M)	11	10 (91 %)	1 (9 %)
Clinical Officers (CO)	13	2 (15 %)	10 (77 %) 1 unknown
Medical Assistants (MA)	8	1 (12.5 %)	7 (87.5 %)
Total	84	60 (71%)	24 (29%)

The majority of respondents (69 %) had seriously considered leaving their post as a result of the incident they described. The most common factors reported were related to issues with managers. Themes identified within this category were challenges in the working relationship with managers and lack of managerial support for staff performance and welfare. Underlying these were problems in communication between managers and staff. This paper reports on those respondent’s who felt pushed to the brink of leaving their posts. However, it is worth noting that the remaining participants (31 %) who were demotivated, but had not seriously considered leaving, described similar experiences when they talked about interactions with their managers.

### Challenges in working relationships

Open criticism and a negative, fault-finding attitude were dominant themes in the relationship between management and MLPs. Many respondents were frustrated by management behavior and supervision practices that looked for mistakes and accused them. They felt demotivated and their work performance was affected. *“I don’t work well because I am always in hot soup everyday…Everyday she finds a mistake to shout at me without any proper reason.”* (NMT, 3142) Others reported unwillingness to report for duties because they received threats from their managers each time they made a slight mistake. *“What they look on are the mistakes you do, not the good things you have done.”* (ENM, 4122) This negatively affected morale and for some MLPs meant they had no desire for work.

The hierarchy between management and staff led to incidences of poor treatment, particularly of junior staff and new recruits. This left staff feeling they were not treated like human beings, while other colleagues had been driven to leave. *“People have left the hospital, people have joined NGOs, because of the attitudes towards new recruits…the way they speak and the way they supervise you is more of a picking somebody…or picking on your personal weaknesses…they want to show their superiority by intimidating others.”* (CO, 3032) Others reported incidences of managers shouting at junior staff instead of helping them. This degrading treatment in their interactions with managers included being shouted at in front of colleagues and patients, or being treated harshly, making staff feel their work was not being appreciated *“…because when they shout at me, it’s like I have done nothing…to their patients.”* (NMT, 1031). Others reported how this impacted on retention. *“If these people are really willing to retain their health worker they need to change their attitudes towards their juniors or their subordinates. That is very important.”* (NMT, 3012)

Favoritism by management was reported as a significant demotivating factor. MLPs complained that management showed overt preferences regarding who accessed in-service training (upgrading or refresher courses), even when trainings were not relevant to them. “…*there’s segregation…I have been working here for 4 years and if there are trainings like PMTCT, which most of it is for maternity, they were taking other people whom they know…I have gone only once to attend a course.”* (NMT, 1061) The same was reported for participation in workshops or seminars. “*No, no…I don’t go for workshops. She* [Matron] *doesn’t consider me in any other field. I can say that when I go for training this month I should expect 6 months or more than that to go for another training, yet my fellow friends can go for training every week, and every time the workshops are advertised.”* (NMT, 3142) This unfairness led to frustrations and negatively affected work performance.

Favoritism in the way salary top-up allowances were administered was also reported*.* MLPs complained that these are not paid equally, especially in CHAM hospitals. *“We were told that those who started work earlier will get higher top-up allowances than those who just started. However, the allowances were increased and those who have worked for 2 years received more than those who have worked for many years.”* (NMT, 4073) Unfairness was also described in the unequal disbursement of salary advances or loans, giving the impression that some cadres were less important than others. *“I was looking for a loan to pay for school fees…she* [administrator] *said*…*we do not have the loan to give to people…barely a week* [later] *some clinical officers went there to ask for the loans and she gave them.”* (Community ENM, 4072)

This lack of transparency generated distrust towards management among nurse/midwives, but some staff also described incidents where their leaders openly demonstrated lack of trust in them, affecting their hard working spirit and confidence. Some reported feeling insecure and afraid in case their boss sent bad reports to their employer, which might even lead to their suspension from duty. *“…my job being at stake now. Each time I carry out my job I only think of may be somebody is going to report such a thing. So I am really working under pressure. Working under such circumstances is very dangerous.”* (CO, 3032)

### Lack of managerial support for staff performance and welfare

MLPs were acutely aware of their need for supportive supervision, particularly in maternal care, as *“…labor ward is a delicate area…”* (NMT, 4121) Staff commented on overwhelming workloads in maternity wards and fear of maternal death. These difficulties were most apparent when they found themselves alone on the wards with no one else to help them, or struggling to find senior staff to assist and mentor them. *“So not only when something has happened then the management should come in, but we want them supervising and supporting us always. But because of this maternal death, it’s when they came saying that we didn’t care for this woman…but we were already complaining that this management team isn’t supporting us, so we really felt bad.”* (NMT, 4121) Junior staff also reported that they did not get the necessary support from seniors when problems arose. In addition, some managers were thought to have insufficient appreciation of the particular challenges involved in maternity care. *“This matron is not a midwife so he doesn’t understand what happens in maternity. So mostly…he is fond of pointing out mistakes.” *(RN/M in-charge, 1011)

Respondents described the impact they felt supportive supervision would have across a range of measures. These included identifying weaknesses, facilitating skills improvement and performance, and providing a mechanism for junior staff to learn from more experienced colleagues. *“With good support, then we can be a performer.”*(RN/M, 1011) Others tried to be proactive in patient care but were demoralized when management did not take action on their decisions or requests, or failed to act upon suggestions fast enough to impact on patient outcomes. “*…if I want something to be done to a certain patient, an idea that can benefit the patient, and then management cannot act or provide what is needed, I feel like I have not helped the patient and I am doing nothing.”* (NMT, 1091)

MLPs reported working for many months without hearing from their managers or supervisors on how they were performing. “…*I have worked for 10 months…but they do not say anything like ‘you are doing your work well’ so you don’t know your weaknesses.”* (ENM, 4091) Staff felt they would benefit from being told if they were doing well, instead of their managers just keeping quiet. Others complained that their managers did not recognize or acknowledge good services or performance. They also mentioned the critical impact of not being appreciated by their managers for the work they do, and how motivating it would be to receive even a small recognition of how hard they try in difficult circumstances. *“Everyone needs to be appreciated when you have done a good job.”* (NMT, 4121) Appraisal that only offered negative feedback was reported to have a detrimental effect on performance, leaving some MLPs demotivated and lacking enthusiasm for work. *“…it really affected my performance. I would say for about 2 days I didn’t touch a patient…If you are demotivated, you don’t have a feeling to work and at the end you find out that the patients are the sufferers.”* (RN/M, 3041)

Managers’ failure to consider personal needs left nurses feeling unvalued and unsupported. If staff approached management about problems at home many were rebuffed. Managers would often say that they were not responsible for staff welfare. *“…if my welfare is not taken care of, I don’t think I will be at work place attending to patients while I have a problem at home. I will not work because I want to solve my problem at home. This definitely affects my work performance.”* (CO, 3091)

Many of the concerns reported by MLPs were linked to a perceived lack of management skills, particularly in terms of communication. There were frequent references to the need for open communication and for management to listen to staff concerns, as staff should have a voice. The lack of respectful communication, such as shouting, harshness and lack of attention to staff ideas, was thought to have an impact on nurses’ intentions to leave the facility. *“I have seen some staff running away from the hospital going for another one. Because there has not been good communication or relationship between the boss and the nurse…the way they communicate to the subordinate.”* (RN, 3041) The same individual suggested that, *“*…*Ministry* [of Health] *should make the opportunities to have managers go through such a process…leadership skills, conflict management, team management, these kind of things, they would be helpful.”*

## Discussion

Efforts to improve health service leadership are crucial to the provision of high quality care. Attention must be paid to equip nurse managers with effective leadership skills and tools that would help them to build an effective leadership style appropriate for their contextual environments. However, the findings from this study show a negative picture of the management and staff relationship in Malawian health facilities. Despite considerable evidence on the benefits of transformational leadership, such as staff motivation, stimulating creativity and innovation, improved retention and decreased burnout [41–43], transactional leadership styles and behavior were a common feature of health workers’ narratives. Management by exception, which is significantly correlated with nursing turnover, was commonplace and staff were demoralized by open criticism and fault-finding attitudes. The hierarchical nature of the health care system was reflected in the harsh treatment of junior staff, as well as other disrespectful interactions, such as being shouted at by managers. Many managers were perceived to display a lack of concern for people and demonstrated poor relationship behaviors. It is clear that the current leadership model in Malawi is not based on existing theoretical understandings and causes considerable distress to staff, including pushing them to consider leaving their posts. The MOH needs to urgently reconsider its HRM policy and set in motion focused and explicit efforts to train managers at all levels in the requisite skills, knowledge and, most importantly, attitudes, to support, motivate and engage the health workforce.

Respondents reported unfairness in the way managers interacted with them including overt favoritism, a lack of confidence or trust in staff and unfairness in allocation benefits, such as training or allowances. These were key drivers of staff dissatisfaction and are destructive to the working relationship between manager and staff [32, 44, 45]. They generate a lack of trust that has implications for workplace relationships and organizational commitment [46] and may have consequences for patient care [47]. These findings are consistent with other studies conducted in sub-Saharan Africa, which have shown that health system favoritism enhanced feelings of injustice leading to staff demotivation [44, 48], but add to them by showingthe potential impact on staff attrition. Unless this hidden behavioral aspect of the work environment is addressed, nurses are likely to continue to leave the health sector for more attractive employment opportunities elsewhere. A manager who wants to have an effective and cohesive team needs to be honest, realistic, and fair when it comes to interactions and expectations. This could be achieved by adopting a more transformative leadership style, based on fairness, empathy, trust and empowerment of health workers, to build staff confidence and treat staff equally [22, 49].

Healthy practice environments, where nurses feel respected and valued, positively impact on staff satisfaction, retention and organisational performance [50]. Staff retention improves when staff feels supported by their nurse manager and this is often linked to a manager’s approachability, openness and balance when dealing with problems that arise during work [51]. The lack of positive contributions from nurse managers affects junior staff since they operate in a context where they suffer accusations for making mistakes. This problem has been largely attributed to inadequate supervision and mentoring. Effective clinical learning environments are characterized by their non-hierarchical nature [52, 53] and positive mentoring supervisory relationships [49]. Mentoring promotes interaction between staff and managers and facilitates the acquisition of skills and knowledge. Promoting the growth and development of junior staff is a major issue in light of the Malawi government’s efforts to alleviate the human resources crisis in the health sector. The Emergency Human Resources Program and any further new plans to boost nurse/midwife numbers will be undermined if the growing cohort of newly qualified health professionals remain dissatisfied due to a lack of supervisory support [7] or find themselves without supportive HRM when they reach their posts.

Supervisory support assures health workers of someone who is on their side and can help resolve problems they encounter in their work environment. In Malawi, nurse/midwives are the mainstay of maternal and newborn health care service provision [54]. Supporting their performance and advancing their knowledge and skills is crucial and is one of the strongest predictors of nursing staff satisfaction in the workplace [51]. However, in Malawian health care settings, transactional leadership characteristics and behavior commonly manifest through managers’ minimal support for these cadres, staff reporting lack of feedback on performance, as well as staff concerns, lack of recognition and appreciation of staff, and unequal access to training opportunities that were based on favoritism, rather than need. Supportive leadership is a key element of transformational leadership. A leader should provide practical support for staff and promote development of knowledge, skills and abilities to improve quality of patient care, as well as paying attention to staff concerns and offering helpful, positive feedback on performance and appreciation [22]. Effective leadership skills will have a positive impact on staff morale, job satisfaction and turnover, and in the delivery of safe and high-quality patient care [55]. For supervisory support to improve, training to build the capacity of managers in mentorship, coaching and role modeling is crucial. This would help create a stable and supportive environment for professional growth and development, and encourage junior staff to learn from senior ones [20].

An additional challenge identified by participants was the lack of appreciation or understanding that nurse managers have of the difficulties staff face in their current work environment. The stress of working in maternity wards without adequate management support and management’s failure to take action on requests or make timely decisions on patient care had an impact on staff retention and was reported as a major concern. The importance of supportive leadership behavior for job satisfaction and the intention to stay in nursing has been described previously [56–58]. Stress and leadership factors continue to impact on nursing staff’s turnover intentions [25] while support from supervisors and leaders can have demonstrable effects. These include decreased turnover intent [59], moderation of the stress-satisfaction relationship [60], reduction of emotional exhaustion and buffering of the negative effects of the job environment [61].

Apart from stress and inadequate management support, the experience of maternal and neonatal deaths, or fear of being involved in a maternal death, were reported to be significant factors in staff demotivation and caused some staff to seriously consider leaving [62]. This is a particular concern in the context of extreme staff shortages. Health care managers with effective leadership skills are an essential component of the solution for ending the staff shortage, but explicit attention to training of these cadres in leadership skills and behaviours is currently inadequate. Implementing strategies to ensure effective leadership is paramount. It is crucial that policy makers take steps to develop and promote viable health service leadership, based on existing theoretical understandings and evidence, to achieve the goal of providing quality care for health care consumers especially in resource constrained settings like Malawi.

A core perception of staff in this study was that management did not care about their welfare. Requests for assistance were often rebuffed or some cadres were helped while others were not, leaving some staff feeling unvalued and unimportant. This is typical of a transactional, laissez-faire style of leadership, where leaders demonstrate uncaring attitudes and avoid making decisions or taking any responsibility for staff personal needs [30]. The resource-poor context and low salaries in the Malawian health system force many health care workers to depend on salary advances and loans. Paying attention to how these are awarded is important to improving performance and retention of health workers. Employees in an organization evaluate the extent to which the organization values their contributions or cares about their well-being and is ready to help them. When perceived organizational support is high, employees are happier, more engaged and more committed to the organization [51]. As such, nurse managers need to treat employees fairly with regards to distribution of resources and outcomes since employees constantly check how they are treated in comparison to their colleagues [63]. This is important, especially in the health care context and culture in Malawi, where inter-professional rivalry is a strong feature [64, 65]. It is the comparison with others that leads to a sense of being treated unfairly, suggesting inequity in the distribution of resources and opportunities. The extent to which the nurse manager demonstrates empathy, consideration and ability to meet the needs of the team members has been emphasized as an important element of supportive supervision [66].

### Limitations

The main purpose of this study was to identify tipping points that made participants feel demotivated or seriously think about leaving their jobs. As such, the incidents recounted were necessarily of a negative nature and it may be the case that a different focus would have revealed instances of positive leadership behaviour in this context. Generalizability of the findings is a concern due to uncertainty whether these individual narratives are representative of the larger MLP population. Future research into positive examples of nurse-manager relationships could be used to identify transformational leadership behaviours in this context and to describe factors enabling or blocking this management style.

In Malawi, rural health centres are often understaffed resulting in more severe workloads than in hospitals. However, only a small percentage (10 %) of participants from health centres’ were interviewed in this study and yet their work conditions are different from those working in hospitals. In addition, the topic under discussion was sensitive and some of the MLP were not very comfortable to reveal their concerns to outsiders for fear of reprisals or even risk of losing their job. Others considered this as an opportunity to air their concerns and find solutions to their problems. This put the interviewers in an awkward position since they had to maintain their position and integrity as a researcher and not a counsellor/adviser.

Another limitation was that this study focussed only on views of staff. Nevertheless, similar concerns were voiced across a range of nursing and clinical cadres, enhancing confidence in the trustworthiness of the results and indicating that poor relationships were commonplace. It would, however, be interesting to explore managers’ perceptions of their working relationships with health facility staff. It would also be preferable to triangulate qualitative data on staff allegations of unfair access to training or loans with a review of facility records. However, record keeping and information system challenges in Malawi would have made this impossible at the time the study was undertaken.

## Conclusion

Findings from this qualitative analysis identified several factors that influenced obstetric care providers’ demotivation and intention to leave. The pervasive and negative impact of poor management-staff relations and lack of support for staff performance and welfare were compounded by a lack of transparency in HRM practices, particularly in access to training and resources. A model of transactional leadership behavior prevailed, evidenced by management by exception and lack of feedback or recognition from managers. These factors demotivate health workers and undermine their efforts. The importance of transformational leadership in nurse-manager relationships cannot be overemphasized. A shift to this style of leadership in Malawi is urgently needed in order to improve the working relationships between managers and staff. This will not only improve providers’ job satisfaction and staff retention, but will also ensure efficiency in health care delivery in Malawi.

## Additional file

Additional file 1: Critical Incident Analysis Instrument. (DOC 30 kb)

## Abbreviations

CHAM: Christian health association of Malawi
CIA: Critical incident analysis
CO: Clinical officer
EmOC: Emergency obstetric care
ENM: Enrolled nurse-midwife
HRM: Human resource management
HSSE: Health systems strengthening for equity
MLP: Mid-level providers
MOH: Ministry of health
NMT: Nurse-midwife technician
PMTCT: Prevention of mother to child transmission
RN/M: Registered nurse- midwife

## Footnotes

1 Basic EmOC-administer parenteral antibiotics; administer uterotonic drugs; administer parenteral anticonvulsants for pre-eclampsia and eclampsia; perform manual removal of placenta; perform removal of retained products of conception (e.g., manual vacuum aspiration, dilation and curettage); perform assisted vaginal delivery (e.g., vacuum extractor); perform neonatal resuscitation (with bag and mask). Comprehensive EmOC-all the above, plus perform surgery (e.g., caesarean section); perform blood transfusion.

## References

[1] Palmer D (2006). Tackling Malawi’s Human Resources Crisis. Reprod Health Matters.

[2] Ngoma D (2012). Human Resources for Health (HRH) in Malawi.

[3] National Statistical Office of Malawi (2008). Population and Housing Census.

[4] National Statistical Office of Malawi (2014). Malawi MDG Endline Survey.

[5] Bradley S (2013). District health managers’ perceptions of supervision in Malawi and Tanzania. Hum Resour Health.

[6] Chimwaza W (2014). What makes staff consider leaving the health service in Malawi?. Hum Resour Health.

[7] Manafa O (2009). Retention of health workers in Malawi: perspectives of health workers and district management. Hum Resour Health.

[8] Blantyre M, Ministry of Health (2004). Human Resources in the Health Sector: Towards a Solution. Government of Malawi.

[9] Aitken JM, Kemp J (2003). HIV/AIDS, Equity and Health Sector Personnel in Southern Africa. EQUINET Discussion Paper 12.

[10] Troy P, Wyness L, McAuliffe E (2007). Nurses’ experiences of recruitment and migration from developing countries: a phenomenological approach. Hum Resour Health.

[11] Adams A, Bond S (2000). Hospital nurses’ job satisfaction, individual and organizational characteristics. J Adv Nurs.

[12] Force VM (2005). The relationship between effective nurse management and nursing retention. J Nurs Manag.

[13] Abualrub RF, Alghamdi MG (2012). The impact of leadership styles on nurses’ satisfaction and intention to stay among Saudi nurses. J Nurs Manag.

[14] AL-Hussami M (2008). Study of nurses’ job satisfaction: The relationship to organizational commitment, perceived organizational support, transactional leadership, transformational leadership, and level of education. Eur J Sci Res.

[15] Zaghloul AA, Al-Hussaini MF, Al-Bassam NK (2008). Intention to stay and nurses’ satisfaction dimensions. J Multidiscip Healthc.

[16] Germain PB, Cummings G (2010). The Influence of nurse leadership on nurse performance: a systematic review. J Nurs Manag.

[17] Soto Fuentes P (2014). Skills for nurses in the field of management and administration: Contemporary challenges to the profession. Aquichan.

[18] Chen CHV (2008). The effect of leader-member exchange, trust, supervisor support on organizational citizenship behavior in nurses. J Nurs Res.

[19] Leiter MP, Maslach C (1998). The impact of interpersonal environment on burnout and organisational commitment. J Organ Behav.

[20] Frankel A (2008). What Leadership Styles Should Senior Nurses Develop?. Nurs Times.

[21] Bass BM (1985). Leadership and Performance Beyond Expectations.

[22] West M (2015). Leadership and Leadership Development in Healthcare: The Evidence Base.

[23] Manongi RN, Marchant TC, Bygbjerg IC (2006). Improving motivation among primary health care workers in Tanzania: a health worker perspective. Hum Resour Health.

[24] Mbindyo PM (2009). Developing a tool to measure health worker motivation in district hospitals in Kenya. Hum Resour Health.

[25] Coomber B, Barriball KL (2007). Impact of job satisfaction components on intent to leave and turnover for hospital-based nurses: A review of the research literature. Int J Nurs Stud.

[26] Cowden T, Cummings GG, Profetto-Mcgrath J (2011). Leadership practices and staff nurses’ intent to stay: a systematic review. J Nurs Manag.

[27] McAuliffe E (2009). Understanding job satisfaction amongst mid-level cadres in Malawi: the contribution of organizational justice. Reprod Health Matters.

[28] Rad AMM, Yarmohammadian MH (2006). A study of relationship between managers’ leadership style and employees’ job satisfaction. Leadersh Health Serv.

[29] Avolio BJ, Bass BM, Jung DI (1999). Re-examining the components of transformational and transactional leadership using the Multifactor Leadership Questionnaire. J Occup Organ Psychol.

[30] Casida J, Parker J (2011). Staff nurse perceptions of nurse manager leadership styles and outcomes. J Nurs Manag.

[31] American Association of Critical-Care Nurses (2005). AACN Standards for establishing and sustaining healthy work environments: A journey to excellence. Am J Crit Care.

[32] Blaauw D (2013). Comparing the job satisfaction and intention to leave of different categories of health workers in Tanzania, Malawi, and South Africa. Glob Health Action.

[33] Fogarty L (2014). Job satisfaction and retention of health-care providers in Afghanistan and Malawi. Hum Resour Health.

[34] Mangham L (2007). Addressing the human resource crisis in Malawi’s health sector: employment preferences of public sector registered nurses. ESAU Working Paper.

[35] Maluwa VM (2012). Moral distress in nursing practice in Malawi. Nurs Ethics.

[36] Ministry of Health Malawi (2011). Malawi Health Sector Strategic Plan 2011–2016.

[37] Flanagan JC (1954). The Critical Incident Technique. Psychol Bull.

[38] MacLachlan M, McAuliffe E (1993). Critical incidents for psychology students in a refugee camp. Couns Psychol Q.

[39] NVivo qualitative data analysis Software; QSR International Pty Ltd. Version 8, 2008.

[40] Green J, Thorogood N (2004). Qualitative Methods for Health Research.

[41] Nielsen K (2009). The mediating effects of team and self-efficacy on the relationship between transformational leadership, and job satisfaction and psychological well-being in healthcare professionals: a cross-sectional questionnaire survey. Int J Nurs Stud.

[42] Weberg D (2010). Transformational leadership and staff retention: an evidence review with implications for healthcare systems. Nurs Adm Q.

[43] Willis-Shattuck M (2008). Motivation and retention of health workers in developing countries: a systematic review. BMC Health Serv Res.

[44] Faye A (2013). Developing a tool to measure satisfaction among health professionals in sub-Saharan Africa. Hum Resour Health.

[45] Thompson SA (2004). The top 10 qualities of a good nurse manager. Am J Nurs.

[46] Gilson L (2006). Trust in health care: theoretical perspectives and research needs. J Health Organ Manag.

[47] Gilson L, Palmer N, Schneider H (2005). Trust and health worker performance: exploring a conceptual framework using South African evidence. Soc Sci Med.

[48] Mbindyo PL, Gilson L (2009). Contextual influences on health worker motivation in district hospitals in Kenya. Implement Sci.

[49] Sherman R, Pross E (2010). Growing Future Nurse Leaders to Build and Sustain Healthy Work Environments at the Unit Level. Online J Issues Nurs.

[50] Aiken LH (2002). Hospital nurse staffing and patient mortality, nurse burnout, and job dissatisfaction. JAMA.

[51] Bailey J (2014). The Effects of Hospital Unit Nurse Leaders’ Perceived Follower Support on Nursing Staff Performance Outcomes.

[52] Fretwell JE (1983). Creating a ward learning environment: the sisters’ role. Nurs Times.

[53] Orton HD, Davis BD (1983). Ward learning climate and student nurse response. Research into Nurse Education.

[54] Lobis S (2011). Expected to deliver: alignment of regulation, training, and actual performance of emergency obstetric care providers in Malawi and Tanzania. Int J Gynaecol Obstet.

[55] Givens RJ (2008). Transformational Leadership: The Impact on Organizational and Personal Outcomes. Emerg Leadersh J.

[56] Albaugh J (2003). Keeping nurses in nursing: the profession’s challenge for today. Urol Nurs.

[57] Blanchard K, Waghorn T (1997). Mission Possible.

[58] Taunton RL (1997). Manager leadership and retention of hospital staff nurses. West J Nurs Res.

[59] Delobelle P (2011). Job satisfaction and turnover intent of primary healthcare nurses in rural South Africa: a questionnaire survey. J Adv Nurs.

[60] Abualrub RF, Omari FH, Abu A, Rub AF (2009). The moderating effect of social support on the stress-satisfaction relationship among Jordanian hospital nurses. J Nurs Manag.

[61] Constable JF, Russell DW (1986). The effect of social support and the work environment upon burnout among nurses. J Hum Stress.

[62] Bradley S (2015). Too few staff, too many patients: a qualitative study of the impact on obstetric care providers and on quality of care in Malawi. BMC Pregnancy Childbirth.

[63] Folger R, Konovsky MA (1989). Effects of procedural justice on reactions to pay decisions. Acad Manag Rev.

[64] Bradley S, McAuliffe E (2009). Mid-level providers, in emergency obstetric and newborn health care: factors affecting their performance and retention within the Malawian health system. Hum Resour Health.

[65] McAuliffe E (2009). Measuring and managing the work environment of the mid-level provider-the neglected human resource. Hum Resour Health.

[66] McGilton KS (2009). Development and psychometric testing of the supportive supervisory scale. J Nurs Scholarsh.

