# Supplementary material for: Working relationships between obstetric care staff and their managers: a critical incident analysis
Source: BMC Health Serv Res. 2016 Aug 26;16(1):441. doi: 10.1186/s12913-016-1694-x (PMC5000514; doi:10.1186/s12913-016-1694-x)
Supplement: Additional file 1: — Critical Incident Analysis Instrument. (DOC 30 kb) [file 12913_2016_1694_MOESM1_ESM.doc]

**Additional file 1. Critical Incident Analysis Instrument**

1. In the past 3 months has there ever been a time when something happened to make you feel dissatisfied or demotivated in your job?

*Describe this event and how you were feeling within yourself*

1. What other reasons led up to this/How did this come about?
2. What were the specific issues in your work that caused you to feel demotivated?

*Prompt for any particular challenges that make it hard for them to feel motivated or do their work*

1. How did you deal with the situation? What did you do to make yourself feel better?
2. If this happened again, how do you think you would react?
3. Are the concerns that caused you to become demotivated still present in your work?

*Refer back to the concerns that were mentioned in describing the event*

1. Do you think they influence your performance in your job? How?
2. How do you manage despite these problems?
3. In the past 3 months has there ever been a time when something happened to make you seriously consider leaving your job?

*Prompt for thoughts of transferring to another facility*

***(if answer is no, finish interview here)***

1. Describe this event:

***(if event described is same as in 2 above skip to question 16)***

1. What were you feeling within yourself?
2. What led up to this/How did this come about?
3. What were the particular issues in your work that caused you to think about leaving?
4. If this happened again, how do you think you would react?
5. Why did you decide against leaving? (or transferring to another facility)
6. What factors helped you to decide to stay?

*If ‘no chance to transfer’ ask what they would do if they got the chance*

1. Are the concerns that caused you to think about leaving/transferring still present in your work?
2. These days, do you often think about leaving/transferring your job?
3. Do you think you will actually leave/transfer?
4. If you do leave/transfer, will you seek employment with another health organisation?

*Where, what type of organisation, what department/cadre?*

1. If you do leave, will you seek employment in another profession?

*Which profession, why, where*

1. How do you think you would feel about leaving your current employer/facility? Why?
2. How do you think you would feel about leaving your current profession? Why?

**Is there anything else you wish to add or other comments you would like to make?**

**Thank you for participating in this interview.**
